# Supplementary material for: An oocyte meiotic midbody cap is required for developmental competence in mice
Source: Nat Commun. 2023 Nov 16;14:7419. doi: 10.1038/s41467-023-43288-x (PMC10654508; doi:10.1038/s41467-023-43288-x)
Supplement: Supplementary file 1 — Supplementary Information [file 41467_2023_43288_MOESM1_ESM.pdf]

An oocyte meiotic midbody cap is required for developmental competence in mice

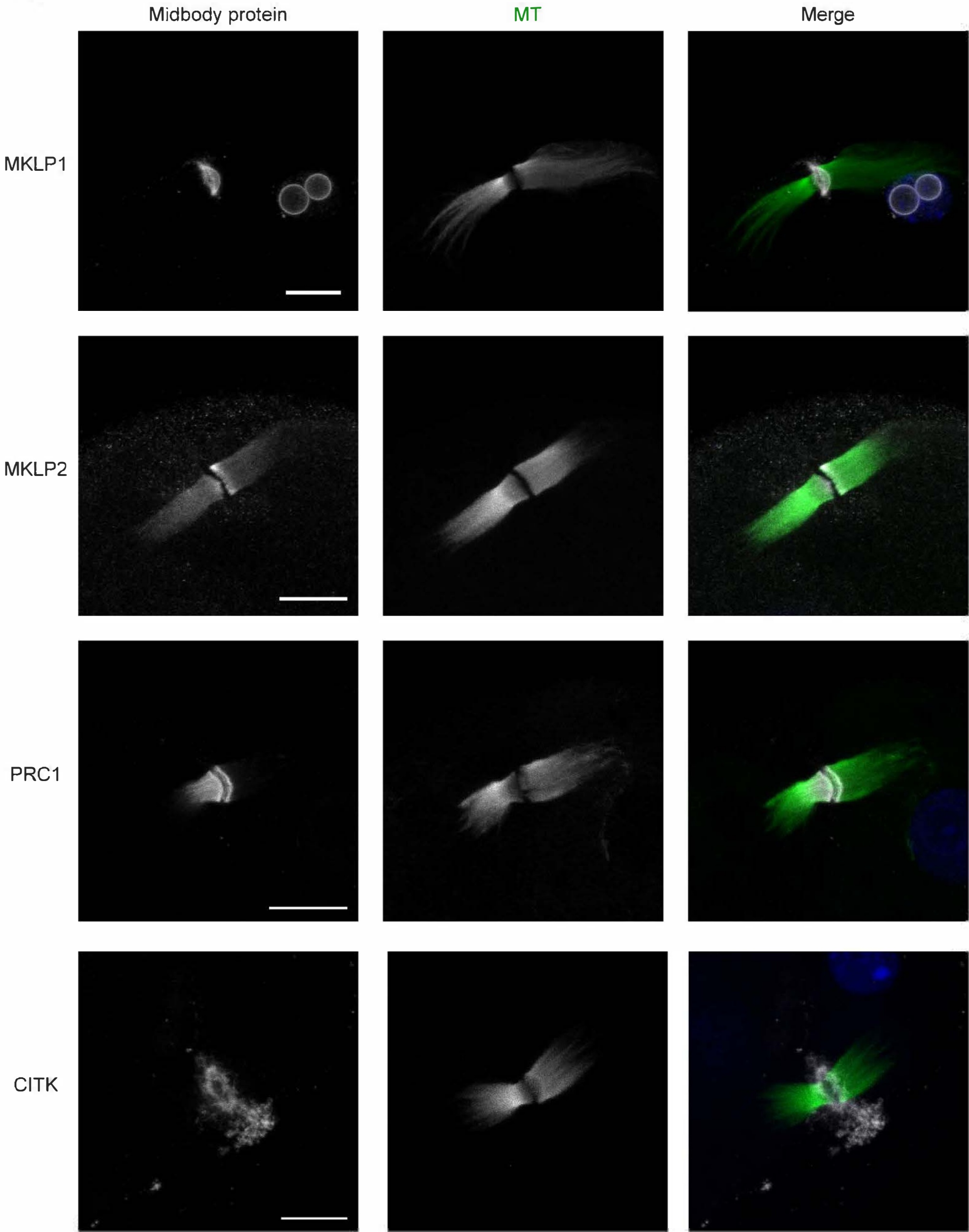
Midbody protein
MT
Merge
MKLP1
MKLP2
PRC1
CITK

Supplementary Figure 1. **Localization of midbody markers in Telophase of meiosis II.** Representative confocal images showing the localization of MKLP1, MKLP2, PRC1, and CITK (gray) relative to midzone spindle (green; tubulin) in midbody from strontium chloride-activated eggs; DAPI in blue. Decondensed DNA can be observed with MKLP1 localization as two circles around the nucleoli. These experiments were replicated 3 times with a total of at least 30 eggs per midbody protein. Scale bars = 10 µm.

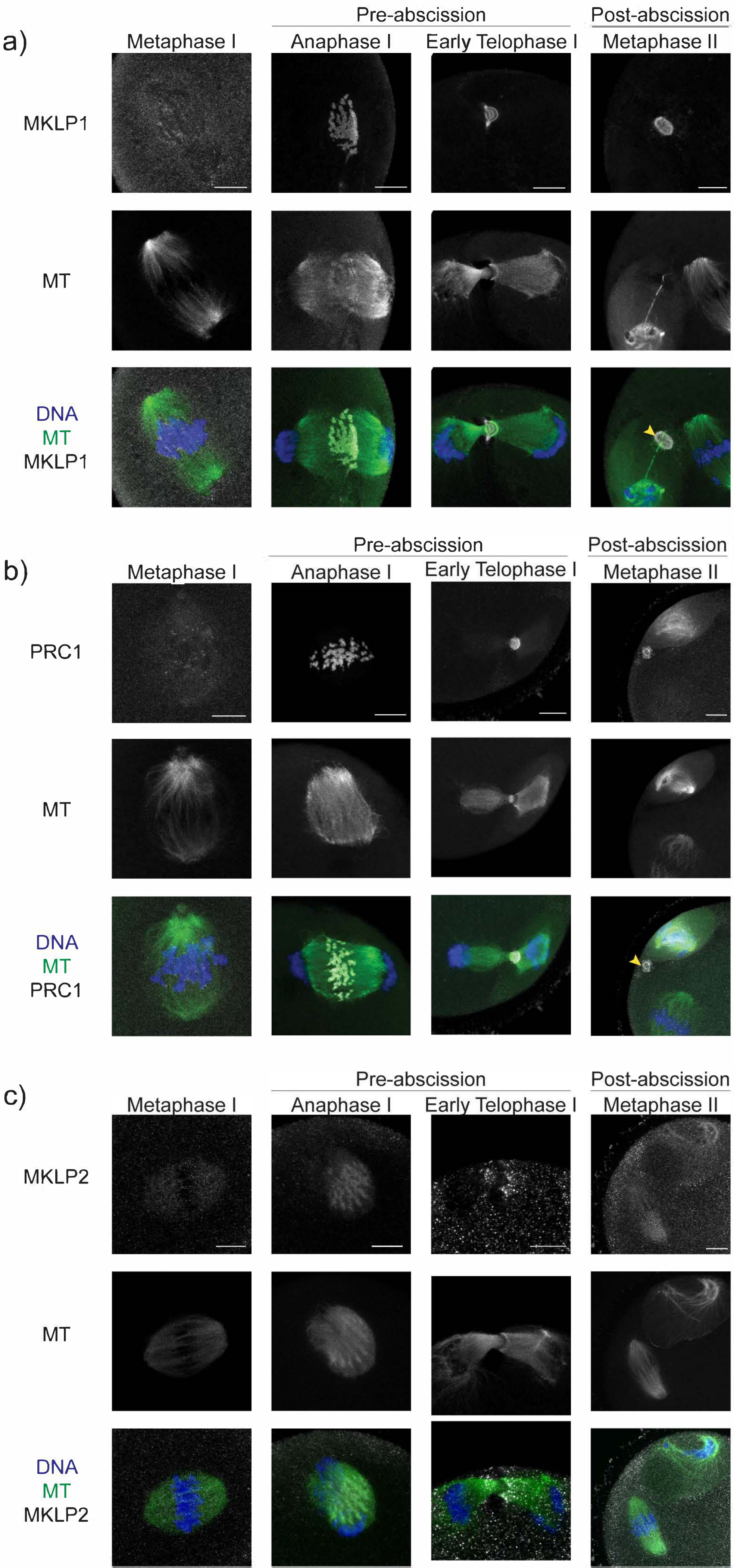

a)
Pre-abscission
Post-abscission
Metaphase I
Anaphase I
Early Telophase I
Metaphase II
MKLP1
MT
DNA
MT
MKLP1
b)
Pre-abscission
Post-abscission
Metaphase I
Anaphase I
Early Telophase I
Metaphase II
PRC1
MT
DNA
MT
PRC1
c)
Pre-abscission
Post-abscission
Metaphase I
Anaphase I
Early Telophase I
Metaphase II
MKLP2
MT
DNA
MT
MKLP2

Supplementary Figure 2. **Midbody protein localization during Meiosis I and at Metaphase of Meiosis II.** a-c) Representative confocal images showing the localization of MKLP1 (a), PRC1 (b), and MLKP2 (c) at Metaphase I, Anaphase I, Telophase I, and Metaphase II; microtubules (gray/green in merge; tubulin) and DNA (blue; DAPI). Yellow arrowheads in (a) and (b) indicate meiotic midbody remnant post-abscission at Metaphase II. These experiments were replicated 3 times with a total of at least 30 eggs per midbody protein and time point. Scale bars = 10 µm.

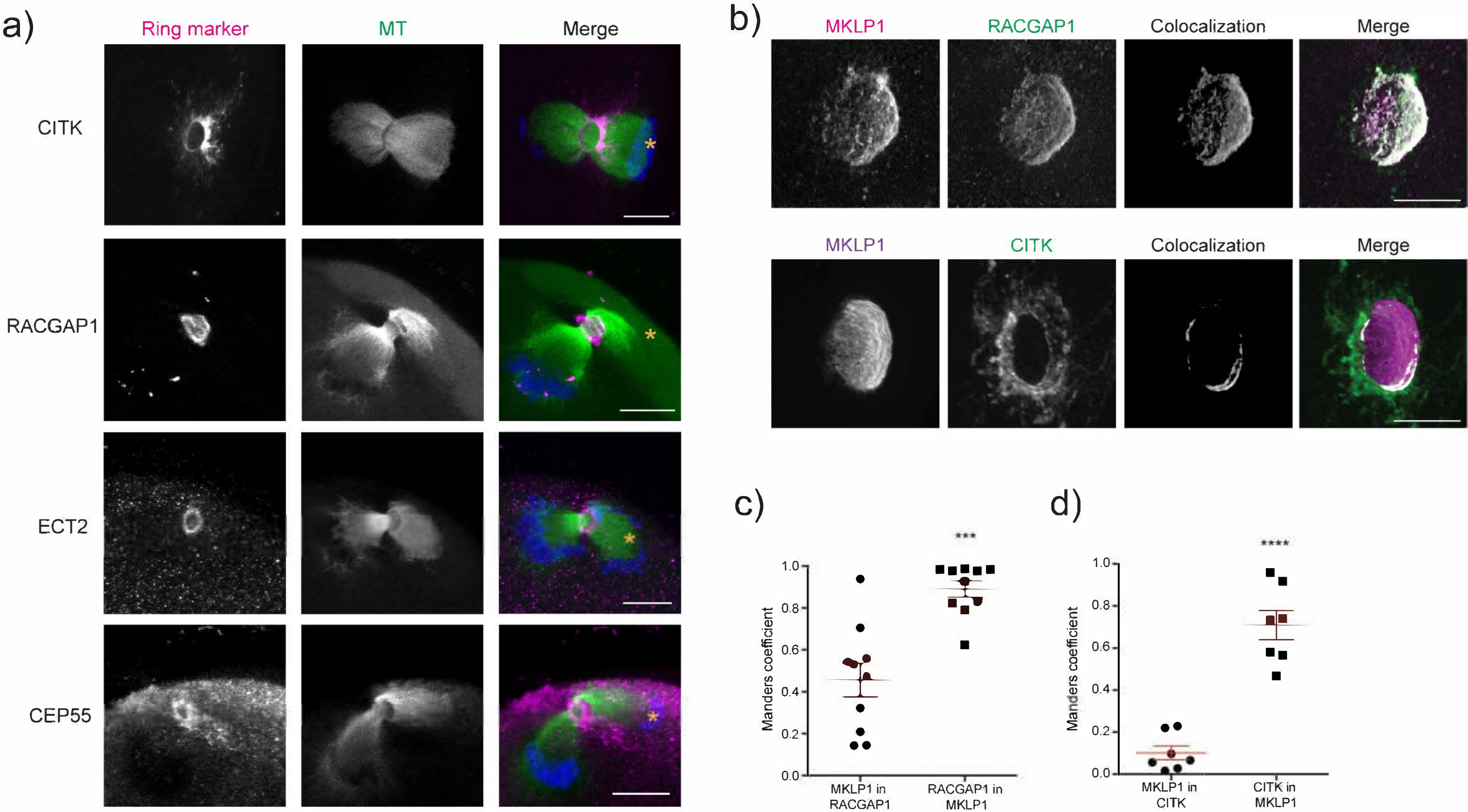

a)
Ring marker
MT
Merge
CITK
RACGAP1
ECT2
CEP55
b)
MKLP1
RACGAP1
Colocalization
Merge
MKLP1
CITK
Colocalization
Merge
c)
***
Manders coefficient
1.0
0.8
0.6
0.4
0.2
0.0
MKLP1 in RACGAP1
RACGAP1 in MKLP1
d)
****
Manders coefficient
1.0
0.8
0.6
0.4
0.2
0.0
MKLP1 in CITK
CITK in MKLP1

Supplementary Figure 3. Meiotic midbody cap composition. a) Representative confocal images for localization of CITK (magenta; first row), RACGAP1 (magenta; second row), ECT2 (magenta; third row), and CEP55 (magenta; fourth row) relative to microtubules (green; tubulin). The asterisk denotes polar bodies. b) Representative STED images comparing the localization of MKLP1 (magenta) with additional ring markers (green; RACGAP1 (top panels) and CITK (bottom panels)). Signal that colocalized between the two ring components compared is shown in gray. b-d) Quantification of Manders coefficient to compare signal colocalization between (c) MKLP1 and RACGAP1, and (d) MKLP1 and CITK. Unpaired Student's t-test, two-tailed; 10 oocytes for (c), 7 oocytes for (d). These experiments were replicated 3 times. Data are presented as mean values +/- SEM. Scale bars = 10µm and 5µm in zoom panels; ***p = 0.0001, ****p<0.0001.

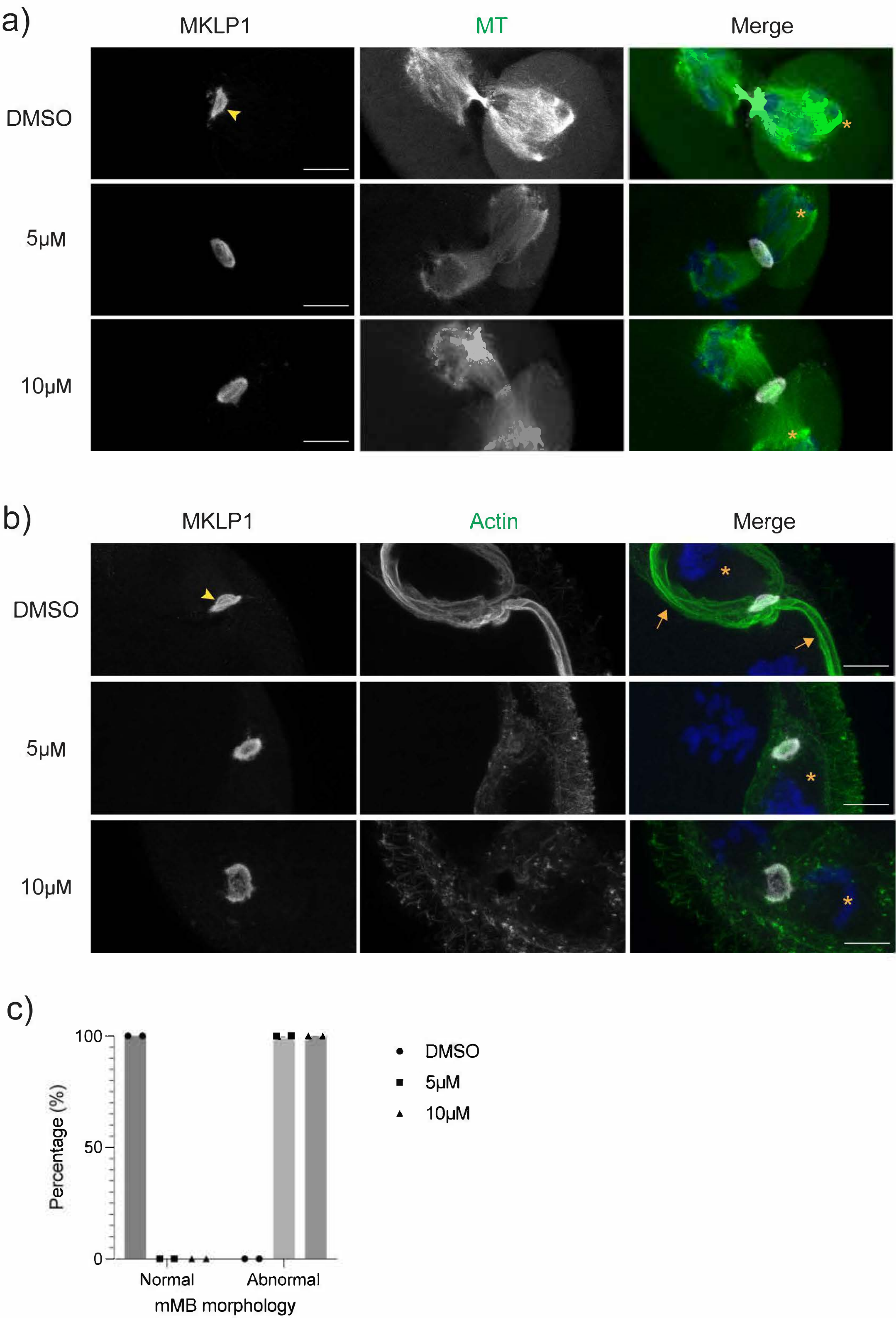
a)
MKLP1
MT
Merge
DMSO
5μM
10μM
b)
MKLP1
Actin
Merge
DMSO
5μM
10μM
c)
100
50
0
Percentage (%)
Normal
Abnormal
mMB morphology
DMSO
5μM
10μM

Supplementary Figure 4. Latrunculin A treatment depolymerizes actin and perturbs microtubules. a-b) Representative confocal images showing the morphology of MKLP1 (gray) relative to midzone spindle (tubulin) (a) and actin (b) (green) in midbodies from oocytes treated with DMSO, or 5 µM and 10 µM of latrunculin A. Orange arrows in (b) highlight the organized localization of actin in the egg and PB. c) Quantification of percentages of normal and abnormal mMBs from oocytes treated with DMSO, or 5 µM and 10 µM latrunculin A. The asterisk denotes polar bodies (PB), and the yellow arrowheads point to the mMB cap present in DMSO groups. These experiments were replicated 3 times with a total of at least 50 eggs per treatment. Scale bars = 10µm.

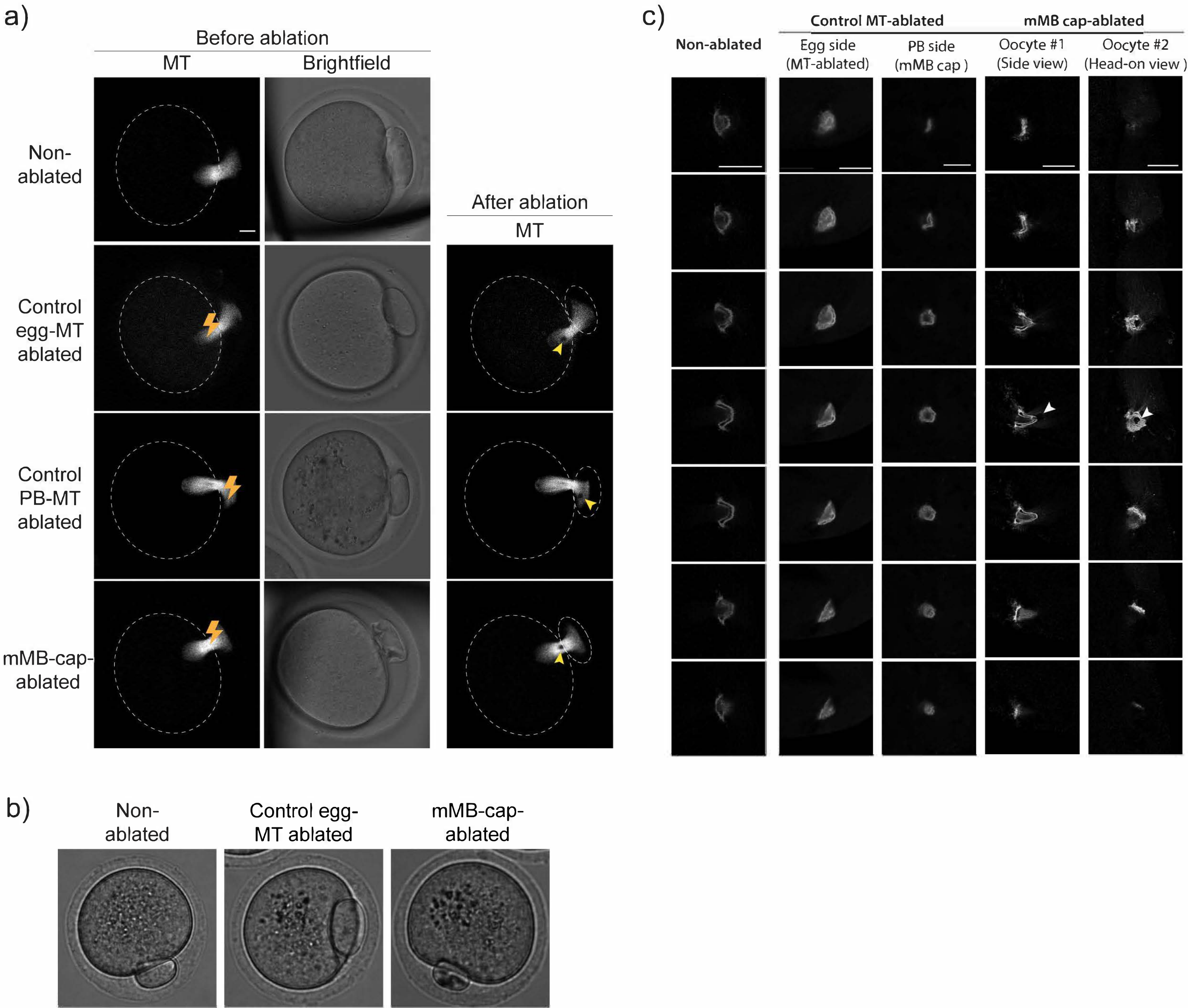
a)
Before ablation
MT
Brightfield
Non-ablated
Control egg-MT ablated
Control PB-MT ablated
mMB-cap-ablated
After ablation
MT
b)
Non-ablated
Control egg-MT ablated
mMB-cap-ablated
c)
Non-ablated
Control MT-ablated
mMB cap-ablated
Egg side (MT-ablated)
PB side (mMB cap)
Oocyte #1 (Side view)
Oocyte #2 (Head-on view)

Supplementary Figure 5. Confirmation of laser ablation. a) Representative confocal images showing microtubules labeled with SiR-Tubulin (gray) and brightfield images of early Telophase I-oocytes for non-ablated, control MT-ablated (egg and polar body sides (PB)), and mMB cap-ablated cells. The area of ablation is marked with a yellow lightning symbol, and the zone after ablation is marked with a yellow arrowhead. The dotted white lines outline the oocyte and the polar body. b) Representative brightfield images of eggs with fully formed polar bodies from non-ablated, control egg side MT-ablated, and mMB cap-ablated oocytes. c) Confocal z series of oocytes from Fig. 6a. These experiments were replicated 4 times with a total of at least 40 oocytes per condition. Scale bars = 10 µm.

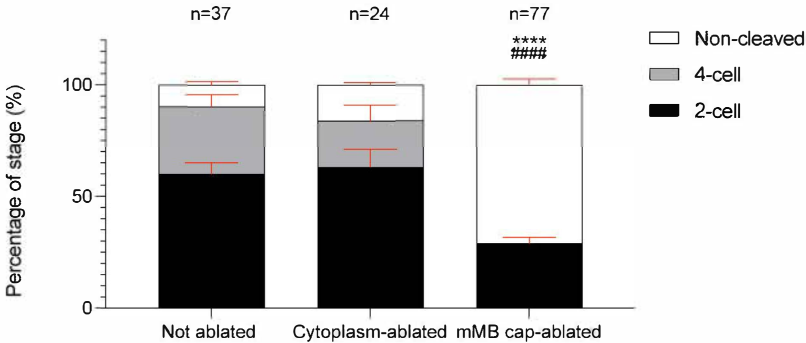

n=37
n=24
n=77
****
####
Non-cleaved
4-cell
2-cell
Percentage of stage (%)
100
50
0
Not ablated
Cytoplasm-ablated
mMB cap-ablated

Supplementary Figure 6. Laser ablation of mMB cap but not cytoplasm leads to embryonic cleavage defects. Quantification of the percentage of parthenotes at each developmental stage. ****$p < 0.0001$ mMB cap-ablated, non-cleaved cells compared to non-ablated group; #### $p < 0.0001$ mMB cap-ablated, non-ablated cells compared to control-ablated group. Tukey's multiple comparisons test; 3 replicates, 30 oocytes.
