# Supplementary material for: An oocyte meiotic midbody cap is required for developmental competence in mice
Source: Nat Commun. 2023 Nov 16;14:7419. doi: 10.1038/s41467-023-43288-x (PMC10654508; doi:10.1038/s41467-023-43288-x)
Supplement: Supplementary file 3 — Description of Additional Supplementary Files [file 41467_2023_43288_MOESM3_ESM.pdf]

Description of Additional Supplementary Files

File Name: Supplementary Movie 1
Description: Dynamic localization of MKLP1 during cytokinesis of meiosis I relative to midzone spindle. Representative live-cell confocal imaging of oocyte in Metaphase I undergoing cytokinesis, overexpressing MKLP1-GFP (magenta) and incubated in SiR-tubulin for spindle visualization (green). This experiment was conducted 3 times with a total of 20 oocytes. Scale bar = 10 µm.

File Name: Supplementary Movie 2
Description: Dynamic localization of MKLP1 during cytokinesis and abscission of meiosis I relative to membrane. Representative live-cell confocal imaging of oocyte in Metaphase I undergoing cytokinesis, overexpressing MKLP1-mCherry (magenta) and GAP43-GFP for membrane visualization (gray). This experiment was conducted 3 times with a total of 20 oocytes. Scale bar = 10 µm.

File Name: Supplementary Movie 3
Description: Symmetric division leads to loss of mMB asymmetry. Animated 3D reconstruction of confocal images of mMB in oocytes compressed during cytokinesis of meiosis I, showing microtubules (green), MKLP1 (gray) and DNA (blue; DAPI). This experiment was conducted 3 times with a total of 30 oocytes

File Name: Supplementary Movie 4
Description: EB3-GFP is more abundant on the egg side than the polar body side. Representative live-cell confocal imaging of oocyte in Telophase I expressing EB3-GFP. This experiment was conducted 3 times with a total of 20 oocytes.

File Name: Supplementary Movie 5
Description: Nascent translation remains on the egg side of the meiotic midbody cap. Animated 3D reconstruction of confocal images of mMB from oocyte showing translation localization (gray; HPG) and MKLP1 (magenta). 3D reconstruction is sectioned along the x and y planes. This experiment was conducted 3 times with a total of 60 oocytes.

File Name: Supplementary Movie 6
Description: Nascent translation leaks into the polar body side when the meiotic midbody cap is disrupted. Animated 3D reconstruction of confocal images of mMB from oocyte treated with 50 µM nocodazole showing translation localization (gray; HPG) and MKLP1 (magenta). 3D reconstruction is sectioned along the y plane. This experiment was conducted 3 times with a total of 40 oocytes.
