# Supplementary material for: An oocyte meiotic midbody cap is required for developmental competence in mice
Source: Nat Commun. 2023 Nov 16;14:7419. doi: 10.1038/s41467-023-43288-x (PMC10654508; doi:10.1038/s41467-023-43288-x)
Supplement: Supplementary file 10 — Reporting Summary [file 41467_2023_43288_MOESM10_ESM.pdf]

nature portfolio

Corresponding author(s): Karen Schindler

Last updated by author(s): 2023-20-10

Reporting Summary

Nature Portfolio wishes to improve the reproducibility of the work that we publish. This form provides structure for consistency and transparency in reporting. For further information on Nature Portfolio policies, see our Editorial Policies and the Editorial Policy Checklist.

Statistics

For all statistical analyses, confirm that the following items are present in the figure legend, table legend, main text, or Methods section.

n/a	Confirmed	
[ ]	[x]	The exact sample size (*n*) for each experimental group/condition, given as a discrete number and unit of measurement
[ ]	[x]	A statement on whether measurements were taken from distinct samples or whether the same sample was measured repeatedly
[ ]	[x]	The statistical test(s) used AND whether they are one- or two-sided<br>*Only common tests should be described solely by name; describe more complex techniques in the Methods section.*
[x]	[ ]	A description of all covariates tested
[x]	[ ]	A description of any assumptions or corrections, such as tests of normality and adjustment for multiple comparisons
[ ]	[x]	A full description of the statistical parameters including central tendency (e.g. means) or other basic estimates (e.g. regression coefficient) AND variation (e.g. standard deviation) or associated estimates of uncertainty (e.g. confidence intervals)
[ ]	[x]	For null hypothesis testing, the test statistic (e.g. *F*, *t*, *r*) with confidence intervals, effect sizes, degrees of freedom and *P* value noted<br>*Give P values as exact values whenever suitable.*
[x]	[ ]	For Bayesian analysis, information on the choice of priors and Markov chain Monte Carlo settings
[x]	[ ]	For hierarchical and complex designs, identification of the appropriate level for tests and full reporting of outcomes
[x]	[ ]	Estimates of effect sizes (e.g. Cohen's *d*, Pearson's *r*), indicating how they were calculated

Our web collection on statistics for biologists contains articles on many of the points above.

Software and code

Policy information about availability of computer code

Data collection	Leica Application Suite X
Data analysis	FIJI (2.3.0/1.53f51), Bitplane Imaris (9.9.1), and GraphPad Prism (9.4.1)

For manuscripts utilizing custom algorithms or software that are central to the research but not yet described in published literature, software must be made available to editors and reviewers. We strongly encourage code deposition in a community repository (e.g. GitHub). See the Nature Portfolio guidelines for submitting code & software for further information.

Data

Policy information about availability of data

All manuscripts must include a data availability statement. This statement should provide the following information, where applicable:

- Accession codes, unique identifiers, or web links for publicly available datasets
- A description of any restrictions on data availability
- For clinical datasets or third party data, please ensure that the statement adheres to our policy

No sequence or proteomic data has been generated in this study. All data supporting the findings of this study are available from the corresponding author upon request. Image files are available upon request because they are many large files in a unique format from the microscope software.

Human research participants

Policy information about studies involving human research participants and Sex and Gender in Research.

Reporting on sex and gender	N/A
Population characteristics	N/A
Recruitment	N/A
Ethics oversight	N/A

Note that full information on the approval of the study protocol must also be provided in the manuscript.

Field-specific reporting

Please select the one below that is the best fit for your research. If you are not sure, read the appropriate sections before making your selection.

- [x] Life sciences
- [ ] Behavioural & social sciences
- [ ] Ecological, evolutionary & environmental sciences

For a reference copy of the document with all sections, see nature.com/documents/nr-reporting-summary-flat.pdf

Life sciences study design

All studies must disclose on these points even when the disclosure is negative.

Sample size	No sample-size calculation was performed for this study. Experimental sample size was determined based on previous studies and experience, consistent across the field (Blengini et al., 2022; Blengini et al., 2021; Aboelenain M and Schindler K., 2021; Yang CR, et al., 2020; Cheng S., et al., 2022). We have found that these sample sizes are sufficient to ensure reproducibility and statistical rigor.
Data exclusions	No data were excluded from the analyses.
Replication	All experiments were replicated 3 to 4 times to confirm reproducibility. In addition, 2 mice were used in each replicate to take into account potential biological variability. We observed that the data were successfully replicated and show consistency.
Randomization	All mice used for the experiments in this studied were purchased from an external vendor. Cells were randomly distributed to control and treated groups.
Blinding	There was no blinding procedure applied because cells were divided into groups prior to collection and analysis. In addition, all steps of the experiments (data collection and analysis) were carried out by the same researcher.

Reporting for specific materials, systems and methods

We require information from authors about some types of materials, experimental systems and methods used in many studies. Here, indicate whether each material, system or method listed is relevant to your study. If you are not sure if a list item applies to your research, read the appropriate section before selecting a response.

Materials & experimental systems

n/a	Involved in the study	
☐	☒	Antibodies
☒	☐	Eukaryotic cell lines
☒	☐	Palaeontology and archaeology
☐	☒	Animals and other organisms
☒	☐	Clinical data
☒	☐	Dual use research of concern

Methods

n/a	Involved in the study	
☒	☐	ChIP-seq
☒	☐	Flow cytometry
☒	☐	MRI-based neuroimaging

Antibodies

Antibodies used	Primary antibodies used were:<br>ECT2 (Fortis Life Sciences, A302-348A) Lot #3<br>CEP55 (Proteintech, 23891-1-AP)<br>PRC1 (Proteintech, 15617-1-AP)

CIT-K (BD Biosciences, 611376)
RACGAP1 (Santa Cruz, sc-271110) Lot# A2518
MKLP1 (Novus Biologicals, NBP2-56923) Lot #R99141
MKLP2 (Proteintech, 67190-1)
RPS3 (Cell Signaling Technology, 2579S) Lot #3
RPS6 (Santa Cruz, sc-74459) Lot # E0820
RPS14 (Proteintech, 16683-1-AP)
RPL24 (ThermoFisher, PA5-62450) Lot # VJ3112597A
CHMP4B (Proteintech, 13683-1-AP)
alpha-tubulin conjugated Alexa Fluor 488 (rabbit, Cell Signaling Technology, Lot #7 5063S; mouse, Invitrogen, Lot # YD370888 322588).

Secondary antibodies used were purchased from Life Technologies:
Anti-rabbit Alexa Fluor 488 (A11029)
Anti-mouse Alexa Fluor 488 (A10042)
Anti-rabbit Alexa Fluor 568 (A10042)
Anti-mouse Alexa Fluor 568 (A10037)
Anti-rabbit Alexa Fluor 633 (A21070)
Anti-mouse Alexa Fluor 633 (A21050)
Anti-rabbit Atto 647N (40839)
Anti-mouse Alexa Fluor 594 (A11032)

Validation	All antibodies were validated by suppliers:<br>ECT2: https://www.fortislife.com/products/primary-antibodies/rabbit-anti-ect2-antibody/BETHYL-A302-348<br>CEP55: https://www.ptglab.com/products/CEP55-Antibody-23891-1-AP.htm<br>PRC1: https://www.ptglab.com/products/PRC1-Antibody-15617-1-AP.htm<br>CIT-K: https://www.bdbiosciences.com/en-eu/products/reagents/microscopy-imaging-reagents/immunofluorescence-reagents/Purified-Mouse-Anti-CRIK.611376<br>RACGAP1: https://datasheets.scbt.com/sc-271110.pdf<br>MKLP1: https://www.novusbio.com/products/mklp1-antibody_nbp2-56923#datasheet<br>MKLP2: https://www.ptglab.com/products/KIF20A-Antibody-67190-1-Ig.htm<br>RPS3: https://www.cellsignal.com/products/primary-antibodies/ribosomal-protein-s3-antibody/2579<br>RPS6: https://datasheets.scbt.com/sc-74459.pdf<br>RPS14: https://www.ptglab.com/products/RPS14-Antibody-16683-1-AP.htm<br>RPL24: https://www.thermofisher.com/antibody/product/RPL24-Antibody-Polyclonal/PA5-62450<br>CHMP4B: https://www.ptglab.com/products/CHMP4B-Antibody-13683-1-AP.htm<br>alpha-tubulin conjugated Alexa Fluor 488 (rabbit: https://www.cellsignal.com/products/antibody-conjugates/a-tubulin-11h10-rabbit-mab-alexa-fluor-488-conjugate/5063 ; mouse: https://www.thermofisher.com/antibody/product/alpha-Tubulin-Antibody-clone-B-5-1-2-Monoclonal/322588).

Animals and other research organisms

Policy information about studies involving animals; ARRIVE guidelines recommended for reporting animal research, and Sex and Gender in Research

Laboratory animals	Outbred, NSA(CF1) female mice (Mus musculus) were used for experiments at 6-8 weeks of age.
Wild animals	The study did not involve wild animals.
Reporting on sex	Only female mice were used for all experiments because of the distinctive features between female and male meiosis.
Field-collected samples	The study did not involve samples collected from the field.
Ethics oversight	All animals were maintained in accordance with the guidelines and policies from the Institutional Animal Use and Care Committee at Rutgers University (Protocol# 201702497) and the Animal Care Quality Assurance at the University of Missouri (Reference# 9695). Temperature was maintained between 70-74F and 50% humidity.

Note that full information on the approval of the study protocol must also be provided in the manuscript.
